# Patient care pathways in acute heart failure and their impact on in-hospital mortality, a French national prospective survey

**DOI:** 10.1016/j.ijcha.2019.100448

**Published:** 2019-12-05

**Authors:** Judith Gorlicki, Marouane Boubaya, Yves Cottin, Denis Angoulvant, Louis Soulat, Sabine Guinemer, Coralie Bloch-Queyrat, Sandrine Deltour, Yves Lambert, Yves Juillière, Frédéric Adnet

**Affiliations:** a Emergency Department, SAMU 93, AP-HP, Avicenne University Hospital, Bobigny, France; b Paris 13 University, Inserm U942, Bobigny, France; c Clinical Research Unit, AP-HP, Avicenne University Hospital, Bobigny, France; d Cardiology Department, University Hospital Center, Dijon, France; e Cardiology Department, Tours University Hospital, EA4245, Loire Valley Cardiovascular Collaboration and FHU SUPORT, Tours University, Tours, France; f Emergency Department, SAMU 35, Pontchaillou University Hospital, Rennes, France; g SAMU 78, Versailles André Mignot Hospital Center, Le Chesnay, France; h Cardiology Department, Nancy-Brabois University Hospital, Institut Lorrain du Cœur et des Vaisseaux, Vandoeuvre-les-Nancy, France

**Keywords:** Acute heart failure, Acute cardiac care, Outcome

## Abstract

**Background:**

Our purpose was to describe the care pathway of patients hospitalized for acute heart failure (AHF) and investigate whether a management involving a cardiology department had an impact on in-hospital mortality.

**Methods:**

Between June 2014 and October 2018, we included patients hospitalized for AHF in 24 French hospitals. Characteristics of the episode, patient’s care pathway and outcomes were recorded on a specific assessment tool. The primary outcome was the association between patient care pathway and in-hospital mortality. The independent association between admission to a cardiology ward and in-hospital mortality was assessed through a multivariate regression model and propensity score matching.

**Results:**

A total of 3677 patients, mean age of 78, were included. The in-hospital mortality rate was 8% (n = 287) and was associated on multivariate regression with advanced age, presence of sepsis, of cardiogenic shock, high New York Heart Association (NYHA) score and increased plasma creatinine level on admission. High blood pressure and admission to a cardiology department appeared as protective factors. After propensity score matching, hospitalization in a cardiology department remained a protective factor of in-hospital mortality (OR = 0.61 [0.44–0.84], p = 0.002).

**Conclusion:**

A hospital course of care involving a cardiology department was associated with an increase in hospital survival in AHF patients. These finding may highlight the importance of collaboration between cardiologists and other in-hospitals specialties, such as emergency physicians, in order to find the best in-hospital pathway for patients with AHF.

Clinical Trial NCT03903198.

## 1 Introduction

Hospitalization for acute heart failure (AHF) is an important event in the patient’s life whether it is the first presentation of heart disease or a decompensation of chronic heart failure [1], [2]. While AHF may lead to a worsened quality of life, increased rates of future hospitalizations or in the worst case death, an episode of AHF may also represents an opportunity to take control of the disease and to optimize treatment [3], [4].

The management of AHF will often involve numerous health care providers, from the first contact with the EMS, the initial care in the emergency department to the physician of the admitting ward. The admitting ward will not always be a cardiology department, but may instead be a geriatrics or general ward, as patients with AHF are increasingly at an advanced age and suffer from substantial comorbidities [5], [6]. In fact, most of AHF patients may not even see a cardiologist during their hospitalization [7].

Whereas the presentation characteristics and outcomes of patients hospitalized for AHF have been described numerous times, knowledge on patients’ in-hospital care pathways and its influence on morbidity and mortality is limited [8], [9], [10], [11]. To study this, a French nationwide survey was set up. As a first step of this prospective observational study, we aimed to describe the different patient care pathways from home to hospital discharge for AHF patients, the treatments received in the different steps of prehospital and in-hospital stays, as well as the outcomes in terms of survival. The primary aim of this study was to assess whether a patient in-hospital care pathway that included a cardiology department (coronary care unit or cardiology ward) decreased in-hospital mortality in AHF patients.

## 2 Methods

### 2.1 Design

Between June 2014 and October 2018, we conducted a national multicentric cohort study in 24 French hospitals. Every physician-staffed Emergency Medical Service (EMS), Emergency department (ED), Coronary Care Unit (CCU) and conventional cardiology ward agreed to participate. A cardiology ward is a hospitalization department where most of the working physicians, including chief department, are cardiologists and where patients are hospitalized for a cardiac acute or chronic pathology. It can be of any size and can include any level of technical platform. A CCU is a cardiology ward where patients’ vitals are continuously monitored. A conventional cardiology ward is a cardiology ward which is not a CCU. In a given hospital, there can be both a conventional cardiology ward and a CCU. Our study is reported in accordance with the STROBE guidelines for the reporting of observational studies [12]. The study was designed in accordance to the Declaration of Helsinski and was approved by the Institutional Review Board.

### 2.2 Selection of participants

All patients above 18 years old that presented with a suspected diagnosis of AHF were prospectively enrolled. AHF was defined following the ESC 2012 Guidelines as the presence of congestive symptoms: cardiac dyspnea, increase of chronic edema, or cardiogenic shock. The diagnosis was established by the first physician in charge of the patient according to those guidelines. The initial treatment at admission was decided by the physician of EMS and/or ED. This treatment could be discussed between the EMS or ED physician and a cardiologist. The admission to a CCU, a conventional cardiology ward or another department was decided by the EMS or ED physician, in accordance with the cardiologist when the patient was admitted in a cardiology ward (CCU conventional). No instruction had been given by the investigator regarding admission criteria to each kind of hospitalization ward. According to French legislation, no written informed consent was required, and the protocol was approved by the national ethics committee (CNIL n°1836586 v 0). All patients were informed of the study plan. No opposition was voiced.

### 2.3 Data collection

Data was collected using a pre-defined assessment tool which followed the patients during their hospitalization. The tool included the baseline characteristics, medical history and previous heart failure events, the first clinical and echocardiography assessment which included the assessment of NYHA and KILLIP scores, the blood test results on admission, as well as the initiated treatments on admission, during hospitalization, and at discharge. The patient care pathways were tracked and registered: the place and time of the first medical contact, the presence of a pre-hospital physician, ED length of stay, place of hospitalization (CCU, cardiology conventional ward, general medicine ward), the kind of specialist involved, place of discharge and in-hospital mortality. The collected data were gathered for analysis by the main investigator and retained for further analysis.

### 2.4 Outcomes

The primary outcome was in-hospital mortality. Secondary outcomes included the complementary clinical exams and the treatments initiated during hospitalization and at discharge. We also aimed to describe the various possible steps of care during the hospitalization.

### 2.5 Statistical analysis

Categorical variables are expressed as numbers (%). Continuous variables are expressed as means (standard deviation, SD), or as medians [25th and 75th percentiles]. We analyzed the association between factors of interest (baseline characteristics, complementary exams, initiated treatments, and wards) and the main outcome, using a chi^2^ test for the qualitative variables, a *t*-test for the quantitative variables and a non-parametric Mann-Whitney test for time variables.

Subsequently, we set up a multivariate logistic regression model to evaluate the independent association between the factors of interest and the main outcome. All the factors associated in univariate analysis with the main outcome with a p value below 0.1 were tested in the model and the selection followed a stepwise procedure.

Finally, we analyzed the association between hospitalization in a CCU and/or cardiology ward with in-hospital mortality using an adjustment with a propensity score to prevent potential confusion bias. The score was estimated using a logistic regression. The primary analyses were based on propensity score matching with a ratio 1:4 and a caliper of 0.05 standard deviation of the logit propensity score. To account for missing data, analyses were conducted using multiple imputations by chained equations with 50 imputations obtained after 10 iterations [13]. The variables considered in the imputation models were all characteristics used in the propensity score, except cardiology stay, which was not imputed. The propensity scores came from 50 independent complete imputed data sets and were averaged and used for matching according “across approach” [14]. Balance in potentials confounders were assessed by standardized mean differences which came from a complete imputed data set [15]. A conditional logistic regression was used to analyze matched data and to estimate the odds ratio (OR) for the relationship between a hospitalization in CCU/ cardiology ward and in-hospital mortality.

Sensitivity analyses were performed using other alternative methods of propensity score analysis. Here we used a matching method with a 1:1 ratio within a caliper of 0.05 standard deviation of the logit propensity score, stratification on the quintiles of the propensity score, and inverse probability of treatment weighting (IPTW). The same analyses were carried out according to the “within approach” [14].

All tests are two-tailed and the results were considered to be statistically significant when p < 0.05. The statistics were performed using R software version 3.3.3 (R foundation for Statistical Computing, Vienna, Austria).

## 3 Results

### 3.1 Patients

Between June 2014 and October 2018, 3677 patients presenting with AHF were included in the study. The mean age was 78 years and 48% were women. Heart failure was previously known in a majority of cases. The main etiology was ischemic cardiopathy (36%) and the main precipitating factor was atrial fibrillation (26%). The clinical presentation was a cardiogenic shock in 109 patients (3%). The left ventricular ejection fraction (LVEF) was reduced (<50%) in more than half of these patients. Diuretics and beta-blockers were the most common medications present on admission. Baseline characteristics are represented in Table 1.

**Table 1:** Patients characteristics.

	Total (n = 3677)	Cardiology admission (n = 2683)	No cardiology admission (n = 756)	p
*Demographic and clinical data*				
Female sex, (%)	1634 (48)	1084 (44.2)	423 (59.7)	<0.0001
Mean (SD) age, years	79 ± 12	76.5 ± 12.6	85 ± 9.7	<0.0001
Mean (SD) BMI, kg/m^2^	27 ± 8	27 ± 7.3	24.8 ± 8.5	<0.0001

*Mean (SD) Blood Pressure, mmHg*				
Systolic	138 ± 31	137.6 ± 31.3	140.6 ± 31.2	0.043
Diastolic	77 ± 18	77.4 ± 18.5	76.3 ± 18.6	0.21
Mean (SD) heartbeat rate, bpm	91 ± 30	91.3 ± 32.5	89.3 ± 23.8	0.089
Previously known heart failure, (%)	2475 (69)	1750 (66.3)	559 (77.3)	<0.0001

*AHF type of presentation*				
Cardiac dyspnea, (%)	3209 (89)	2350 (88.)	653 (89.3)	0.65
Increase of chronic edema, (%)	799 (22)	626 (23.6)	125 (17.1)	0.0002
Cardiogenic shock, (%)	109 (3)	101 (3.8)	6 (0.8)	<0.0001
AHF hospitalization in previous year, (%)	1404 (42)	1018 (41)	302 (44.4)	0.12

*Precipitating factor*				
Atrial arrhythmia, (%)	772 (26)	576 (27.4)	156 (25.7)	0.44
Sepsis, (%)	723 (25)	414 (19.7)	259 (42.7)	<0.0001
High blood pressure, (%)	376 (13)	260 (12.4)	92 (15.2)	0.081
Low compliance to treatment, (%)	226 (8)	183 (8.7)	37 (6.1)	0.047
Ventricular arrhythmia, (%)	48 (1.5)	47 (2.2)	1 (0.2)	0.001
Other (%)	1152 (39)	819 (38.8)	214 (35.1)	0.11
Reduced LVEF < 50%, (%)	631 (59)	583 (62.2)	34 (38.2)	<0.0001
Mean (SD) measured LVEF, %	41 ± 15	40.8 ± 15.2	45.1 ± 15.7	0.063
Mean (SD) plasma creatinine rate, µmol/L	122 ± 69	123.9 ± 68	122.6 ± 73.8	0.71
NT-proBNP > 125 or BNP > 35 pg/mL (%)	2641 (95.6)	1950 (96.8)	520 (90.9)	<0.0001

*NYHA score*				
I, (%)	42 (1.5)	31 (1.6)	6 (1.)	<0.0001
II, (%)	312 (12)	192 (9.8)	86 (18.4)	
III, (%)	913 (35)	712 (36.2)	164 (35.)	
IV, (%)	1307 (51)	1034 (52.)	211 (45.)	
Pathways and lengths of stay				

*First encounter*				
General practitioner, (%)	1153 (34)	858 (33.)	227 (33.5)	**<**0.0001
Physician-staffed EMS, (%)	675 (20)	489 (19.3)	150 (22.1)	
Firemen, ambulance, (%)	318 (9)	241 (9.5)	67 (9.9)	
Relatives, (%)	248 (7)	175 (6.9)	60 (8.8)	
Nurse, (%)	179 (5)	92 (3.6)	67 (9.9)	
Cardiologist, (%)	158 (5)	152 (6)	5 (0.7)	
No call, (%)	699 (20)	530 (20.9)	102 (15)	
Physician-staffed EMS care, (%)	519 (15)	415 (16.5)	78 (10.3)	<0.0001
Median [IQR] time between first symptoms and arrival at hospital, days	2 [0–6]	2 [0–7]	1 [0–3]	0.0004

*Treatment*				
Diuretics (%)	423 (84.7)	1618 (83)	624 (90.8)	<0.0001
Oxygen (%)	2136 (76.6)	1484 (78.1)	531 (79.8)	0.36
NIV (%)	383 (16.1)	287 (17.9)	78 (13.6)	0.022
Nitrates (%)	584 (22.9)	433 (24.7)	120 (20.5)	0.045
Inotrope (%)	54 (2.2)	46 (2.7)	5 (0.9)	0.019

Data in the table are numbers (%) for categorical data and mean ± standard deviation or median [interquartile range] for continuous data depending on the distribution. AHF: acute heart failure, BMI: body mass index, BNP: brain natriuretic protein, b.p.m: beats per minute, CCU: coronary care unit, EMS: emergency medical service, IQR: interquartile range, LVEF: left ventricular ejected fraction, NT-proBNP: N-terminal fragment of brain natriuretic protein, NYHA: New York heart association, SD: standard deviation.

### 3.2 Patient care pathways

In more than 50% of cases the general practitioner or the EMS represented the first encounter with a health care staff. In 9% of cases the patient presented directly at the hospital. The first encounter occurred after an average (median) of one day after the onset of symptoms. Characteristics of the first encounter are presented in Table 1.

**Fig. 1:**
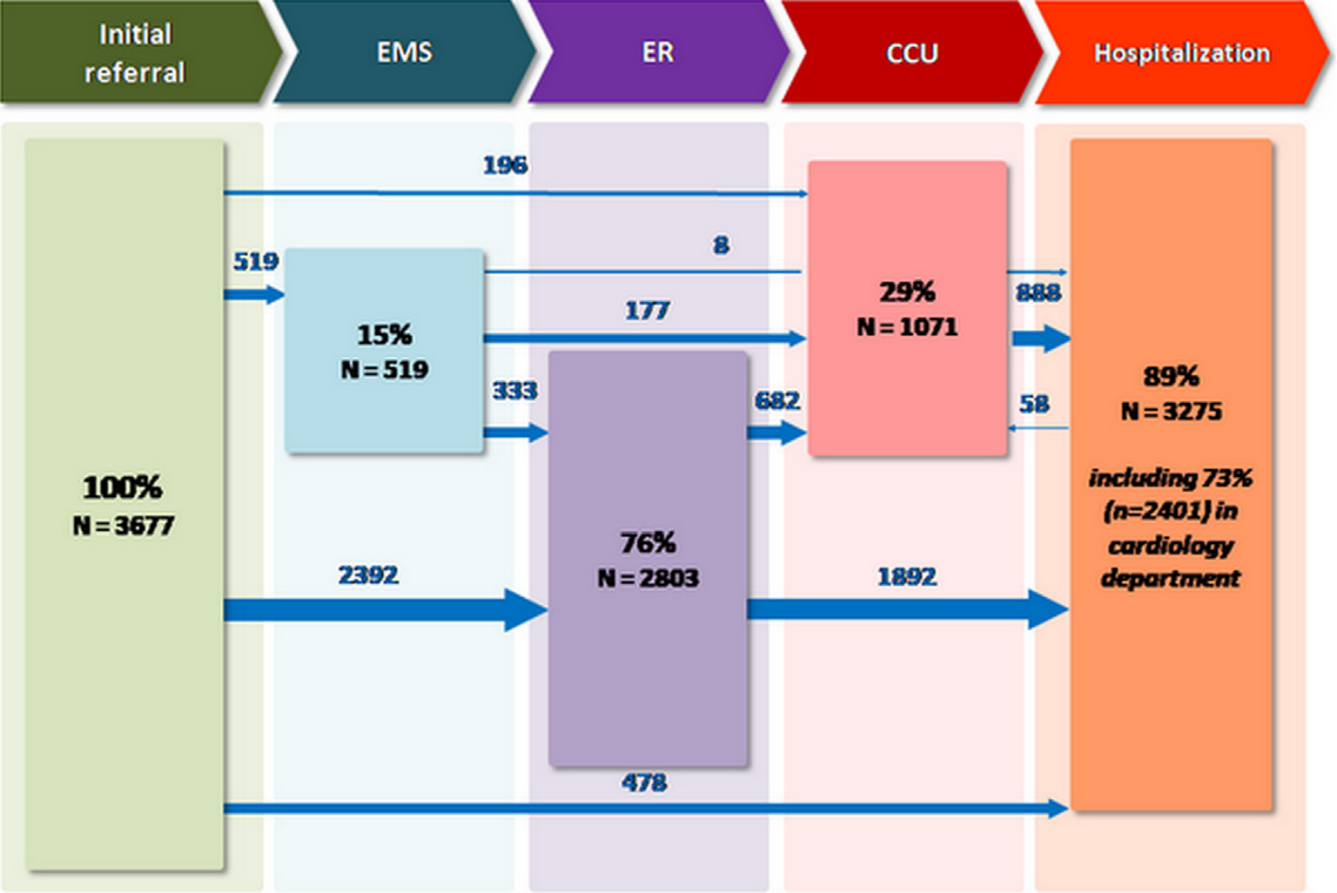
Care pathways of patients with acute heart failure.

The first medical treatment was carried out by a physician-staffed EMS and/or the ED in 80% of patients. Then, only about one third of patients were transferred to the CCU. Later, 2683 (73%) patients were admitted to the CCU and/or cardiology ward. Fig. 1 demonstrates the different pathways. The overall median length of stay in-hospital was eight days. The median length of stay was one hour with physician-staffed EMS, eight hours in the ED, three days in CCU and seven days in a conventional ward (Fig. 1).

At discharge, two-third of the patients went back home. The remaining third were discharged to rehabilitation centers, nursing homes, and other hospitals (Table 1).

### 3.3 Mortality during hospitalization

The in-hospital mortality was 8% (n = 287). On univariate analysis, a significant association was found between increased in-hospital mortality and hospitalization in cardiology (CCU or conventional ward). The other factors significantly associated with in-hospital mortality are listed in Table 2.

**Table 2:** Univariate analysis on in-hospital mortality.

Variables of interest	Not deceased (n = 3390)	Deceased (n = 287)	p-trend
Female sex (%)	1506 (48)	128 (49)	0.89
Mean (SD) age, years	78 ± 12	82 ± 13	<0.0001
Mean (SD) BMI, kg/m^2^	27 ± 8	25 ± 8	0.02

*First encounter*			
General practitioner, (%)	1061 (33)	92 (35)	<0.0001
Cardiologist, (%)	153 (5)	5 (2)	
Nurse, (%)	149 (5)	30 (11)	
No call, (%)	643 (20)	56 (21)	
Firemen, ambulance, (%)	295 (9)	23 (9)	
Relatives, (%)	236 (7)	12 (4)	
Physician-staffed EMS, (%)	627 (20)	48 (18)	
Time between first symptoms and arrival at hospital, median [IQR], days	2 [0–7]	1 [0–5]	0.03
Mean (SD) heartbeat rate, bpm	90 ± (31)	94 ± (29)	0.08
Mean (SD) Systolic blood pressure, mmHg	139 ± (31)	129 ± (30)	<0.0001
Mean (SD) Diastolic blood pressure, mmHg	77 ± (18)	73 ± (17)	0.0004
Mean (SD) LVEF, %	42 ± (15)	36 ± (16)	<0.0001
Reduced LVEF <50%, (%)	575 (58)	36 (16)	0.049
AHF type: Cardiogenic shock, (%)	76 (2)	33 (12)	<0.0001
AHF type: Cardiac dyspnea, (%)	2983 (89)	226 (81)	<0.0001
AHF type: Increase of edema, (%)	742 (22)	57 (20)	0.51
Etiology: Ischemic cardiopathy, (%)	1014 (36)	84 (35.3)	0.92
Etiology: Hypertensive cardiopathy, (%)	638 (23)	42 (18)	0.09
Etiology: Valvopathy, (%)	597 (21)	55 (23)	0.49
Etiology: Rhythmic cardiopathy, (%)	925 (33)	72 (30)	0.48
Etiology: Other, (%)	1062 (39)	67 (28)	0.15
Previously known heart failure, (%)	2283 (69)	192 (69)	0.98
Precipitating factor: sepsis (%)	633 (23)	90 (40)	<0.0001
Precipitating factor: atrial arrhythmia (%)	720 (27)	52 (23)	0.26
Precipitating factor: ventricular arrhythmia (%)	43 (2)	5 (2)	0.41
Precipitating factor: High blood pressure (%)	362 (13)	14 (6)	0.003
Precipitating factor: low compliance to treatment (%)	217 (8)	9 (4)	0.039
Precipitating factor: other (%)	1062 (39)	90 (40)	0.98
NYHA score: IV, (%)	1186 (50)	121 (65)	<0.0001
Mean (SD) admission natremia, mEq/L	138 ± 8	136 ± 11	0.14
Mean (SD) admission creatinine rate, µmol/L	120 ± 66	150 ± 88	<0.0001
NT-proBNP > 125 or BNP > 35 pg/mL (%)	2452 (95.9%)	189 (91.7%)	0.009
AHF hospitalization in the previous year, (%)	1300 (42)	104 (90)	0.52
Physician-staffed EMS care, (%)	474 (15)	45 (16)	0.56
ED admission, (%)	2569 (76)	234 (81)	0.034
Median [IQR] time before ED care, min	42 [17–99]	46 [11–108]	0.68
Contact ED physician and cardiologist, (%)	1784 (71)	140 (61)	0.001
Hospitalization in cardiology, (%)	2508 (79)	175 (64)	<0.0001
Assessment by a cardiologist, (%)	2517 (79)	180 (67)	<0.0001
Treatment: diuretics, (%)	2226 (84.6)	197 (85)	0.87
Treatment: oxygen, (%)	1952 (76)	184 (80)	0.24
Treatment: NIV, (%)	341 (15.7)	42 (21.1)	0.058
Treatment: nitrates, (%)	538 (23)	46 (22)	0.88
Treatment: inotrope, (%)	47 (2)	7 (3)	0.20

Data in the table are numbers (%) for categorical data and mean ± standard deviation or median [interquartile range] for continuous data depending on the distribution. AHF: acute heart failure, BMI: body mass index, bpm: beats per minute, CCU: coronary care unit, ED: emergency department, EMS: emergency medical service, IQR: interquartile range, LVEF: left ventricular ejected fraction, NYHA: New York heart association, SD: standard deviation.

On multivariate analysis, factors associated with increased in-hospital mortality included age (included as a continuous variable. The OR is for a one-year increase) (OR 1.02 [1.01–1.04]), an episode of sepsis (OR 1.73 [1.26–2.33]), a cardiogenic shock (OR 6.41 [3.86–10.56]), a NYHA score equal to four (as a dichotomous factor: 4 or <4) (OR 1.61 [1.17–2.22]) and an elevated creatinine rate (included as a continuous variable. The OR is for a 10 µmol/L increase) (OR 1.05 [1.03–1.06]). The presence of high blood pressure at presentation and hospitalization in a cardiology ward seemed to be protective factors ((OR 0.44 [0.25–0.78]) and OR 0.53 [0.40–0.72] respectively). Results of the multivariate analysis are presented in Table 3.

**Table 3:** Multivariate analysis.

Variables	OR [CI95%]	p
Age^a^	1.02 [1.01–1.04]	0.0004
Cardiogenic shock	6.41 [3.86–10.56]	<0.0001
Sepsis	1.73 [1.26–2.33]	0.0005
NYHA (4 vs < 4)	1.61 [1.17–2.22]	0.004
Precipitating factor: High blood pressure	0.44 [0.25–0.78]	0.005
Creatinine rate^b^	1.05 [1.03–1.06]	<0.0001
Hospitalization in CCU or cardiology ward	0.53 [0.40–0.72]	<0.0001

CCU: coronary care unit, NYHA: New York heart association.

a Included as a continuous variable. The OR is for a one-year increase.

b Included as a continuous variable. The OR is for a 10 µmol/L increase.

**Fig. 2:**
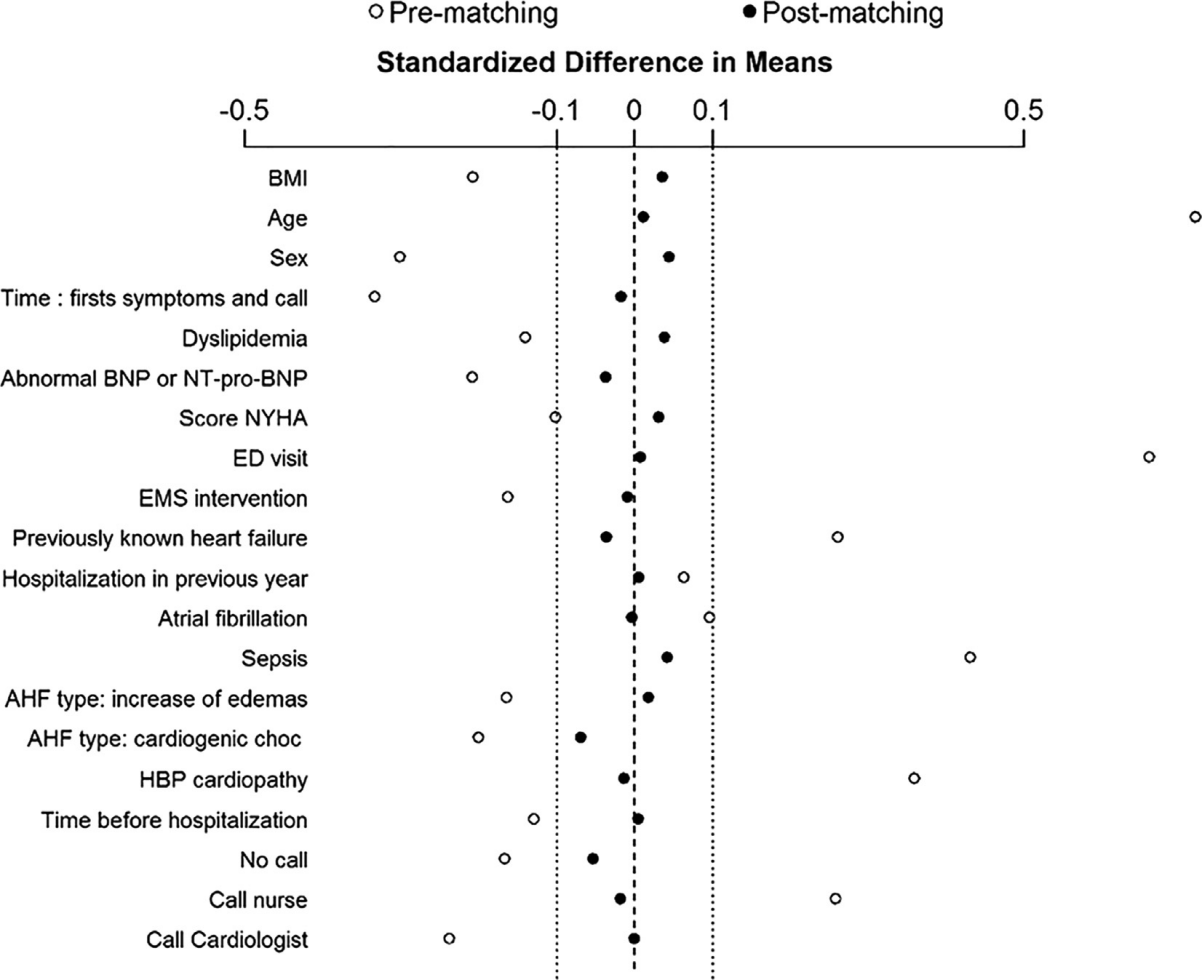
Accuracy of propensity score matching. Propensity score was set up to predict in-hospital mortality. For each variable included in the propensity score, standardized difference in mean between the groups “cardiology admission” and “no cardiology admission” is given before and after matching those groups on the propensity score.

**Fig. 3:**
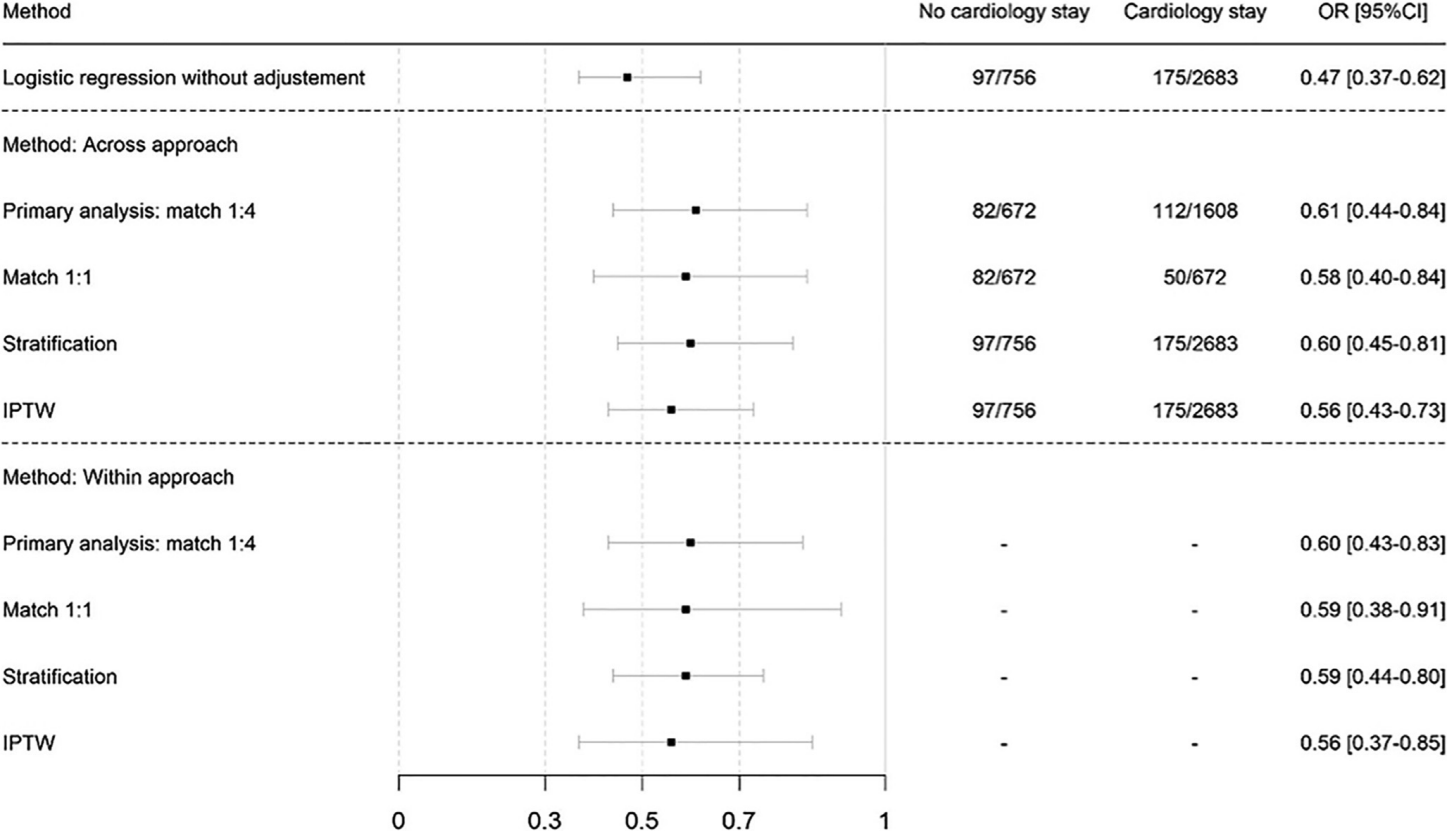
Association between in-hospital mortality and hospitalization in cardiology. First rank is the odd ratio (OR) without adjustment. Second rank is the OR adjusted on propensity score using the across approach method with 4 different matching ratios. Third rank is the OR adjusted on propensity score using the within approach method (sensitivity analysis) with 4 different matching ratios.

The propensity score used to determine the independent association between mortality and hospitalization in the CCU and/or cardiology department included sex, age, BMI, first encounter, cardiogenic shock, increase in edemas, High Blood Pressure (HBP) cardiopathy, sepsis, atrial fibrillation, dyslipidemia, hospitalization for AHF in the previous 12 months, previously known heart failure, being taken care of by physician-staffed EMS, emergency department visit, NYHA score, creatinine rate, abnormal BNP (>35 pg/mL) or NT-pro-BNP (>125 pg/mL), time between first symptoms, and first encounter Fig. 2. When adjusting the on propensity score, a hospitalization in the CCU or Cardiology Department was significantly inversely associated with in-hospital mortality (OR 0.61 [0.44–0.84], p = 0.002), Fig. 3.

## 4 Discussion

This study provides a real-life picture of the patients’ care pathway before and during their hospitalization for AHF in 24 French hospitals. Very few studies have investigated the specific relation between care pathways and mortality.

Our main result clearly shows that admission to a cardiology ward or to CCU was independently and strongly associated with lower in-hospital mortality (OR 0.61 [0.44–0.84]). A national survey conducted in England and Wales in 2008–2009 found a decreasing association between length of stay in specialist services and mortality for AHF patients [7]. Nevertheless, this result was not the primary end-point of their study. Conversely, a recent analysis from the REALITY-AHF registry that included 1,682 AHF patients found no difference in in-hospital mortality between patients managed by emergency physicians and those managed by cardiologists [16].

Several hypotheses could explain this association between improved outcomes following AHF and patient care pathways involving the cardiology or CCU wards. First, this difference may result from a delay in the initiation of treatment which is known to be associated with mortality [17], [18], [19], [20]. One could presume that the number of patients receiving appropriate treatments will be higher for those immediately taken into care by a cardiologist, as described in the REALITY-AHF registry [16]. However in this study and in our cohort, admission to the ED did not appear to be associated with mortality. Another hypothesis is that patients hospitalized in the CCU or cardiology department are more closely monitored, and thus a new cardiologic event or deterioration could be detected earlier than in a general ward. Moreover, all patients hospitalized in a cardiology unit receive early echocardiographic assessment and, likely, more targeted treatment. Patients hospitalized in cardiology department also have their long-term heart failure treatment plan revised by cardiologists, which can have a positive impact on outcome, especially if it is done at the beginning of the hospitalization. For patients with de novo heart failure this assessment constitutes the first investigation of their cardiac pathology and the first prescribed heart failure treatment. In our cohort, 16% of the patients with a de novo acute heart failure were hospitalized in a non-cardiology ward and thus didn’t receive this first assessment which may be even more impacting on outcome. Furthermore, patients hospitalized in cardiology will receive targeted education during hospitalization. However, even if this last factor has an impact on compliance with at-home treatment and on long-term outcome [4], it is unknown whether it impacts in-hospital mortality.

Deciding to admit every patient with AHF to the cardiology department would be unrealistic, due to the increasing number of such patients and because of the necessity of holistic care in patients with one or more important comorbidities. Besides, objective criteria for hospitalization in the CCU or ICU have been established [21]. A more feasible option is a tighter collaboration between cardiologists and other practitioners, which could improve a patient’s outcome when admitted to other wards [22]. In a recent French survey of 132 AHF patients, no association was found between hospitalization in cardiology departments and mortality because of the wide involvement of cardiologists in the management of patients hospitalized in the non-cardiology departments [23]. In our study, the cooperation between Cardiologists and Emergency Physicians appears as a protective factor for in-hospital mortality. This cooperation between specialists should be continued during the entire hospital admission and could take place as a “AHF cardiologist mobile team” as has previously been suggested [23].

Most of the previous studies describing patients hospitalized for AHF focus on the in-hospital course [8], [9], [10], [11]. In 2016, a small cohort study in France described the prehospital and in-hospital care pathways of patients hospitalized for AHF and their treatments and outcomes depending on the departments involved in care [23]. The study was conducted in three hospitals and included only 119 patients [23]. Our study is the first to report on a national scale the step by step patient care pathways from first point of contact to hospital discharge, to associate in-hospital mortality with specific patients’ pathways.

The patients’ characteristics in our cohort are comparable with previous studies. The mean age was 78 years and two patients out of three had a chronic heart failure or previous history of AHF [7], [8], [9], [10], [11], [23]. The main precipitating factors were atrial fibrillation and sepsis as previously described [8], [9], [10], [11], [23], [24]. Furthermore, in-hospital mortality rate was 8%, similar to what it was in previous studies [7], [8], [9], [23] except for EFICA where it was up to 27%, but that study only enrolled patients requiring admission to the ICU and CCU, where outcomes are known to be somewhat worse than the average [11]. These observations reinforce the validity of our cohort.

## 5 Limitations

Our study presents several limitations. First, we focused on in-hospital mortality but did not follow patients after discharge, so we could not assess either re-hospitalization or 30-days, six months or 1-year mortality, which are known to be high after an AHF episode and are widely used as prognostic criteria [7], [23], [25], [26]. Second, diagnosis was made by the physician on field and no independent adjudication have been made. Hence, some patients with another acute pathology than AHF can have been wrongly included in the study, explaining a low BNP or NT-proBNP rate in 4%. However, this situation also occurs in real life, which is what we aimed to describe and analyze. Third, cardiologists were part of each local board of our study, which may have led to a better management of AHF patients than the usual standard of care. However, we were still able to find a difference between patients admitted to a cardiology departments and non-cardiology departments, meaning that AHF management was not optimum for all enrolled patients.

## 6 Conclusion

In-hospital mortality of AHF was significantly lower when the patient care pathway involved admission to a CCU or cardiology ward. Cooperation between general-ward physicians and cardiologists should be reinforced in order to give the same specialized cardiac management to all patients.

## References

[1] Krumholz H.M. (2013). Post-hospital syndrome — an acquired, transient condition of generalized risk. N. Engl. J. Med..

[2] Mesquita E.T., Jorge A.J.L., Rabelo L.M., Souza C.V. (2016). Understanding hospitalization in patients with heart failure. Int. J. Cardiovasc. Sci..

[3] Cowie M.R., Anker S.D., Cleland J.G.F., Felker G.M., Filippatos G., Jaarsma T., Jourdain P., Knight E., Massie B., Ponikowski P., López-Sendón J. (2014). Improving care for patients with acute heart failure: before, during and after hospitalization: Improving care in acute heart failure. ESC Heart Fail..

[4] Juillière Y., Jourdain P., Suty-Selton C., Béard T., Berder V., Maître B., Trochu J.-N., Drouet E., Pace B., Mulak G., Danchin N. (2013). Therapeutic patient education and all-cause mortality in patients with chronic heart failure: a propensity analysis. Int. J. Cardiol..

[5] Kaya H., Yilmaz M.B. (2015). In-hospital journey of patients with heart failure. Int. J. Cardiovasc. Acad..

[6] Kul S., Barbieri A., Milan E., Montag I., Vanhaecht K., Panella M. (2012). Effects of care pathways on the in-hospital treatment of heart failure: a systematic review. BMC Cardiovasc. Disord..

[7] Cleland J.G.F., McDonagh T., Rigby A.S., Yassin A., Whittaker T., Dargie H.J., on behalf of the National Heart Failure Audit Team for England and Wales (2011). The national heart failure audit for England and Wales 2008–2009. Heart.

[8] Fonarow G.C., Corday E. (2004). ADHERE Scientific Advisory Committee, Overview of acutely decompensated congestive heart failure (ADHF): a report from the ADHERE registry. Heart Fail. Rev..

[9] Nieminen M.S., Brutsaert D., Dickstein K., Drexler H., Follath F., Harjola V.-P., Hochadel M., Komajda M., Lassus J., Lopez-Sendon J.L., Ponikowski P., Tavazzi L., on behalf of the EuroHeart Survey Investigators (2006). EuroHeart Failure Survey II (EHFS II): a survey on hospitalized acute heart failure patients: description of population. Eur. Heart J..

[10] Maggioni A.P., Dahlström U., Filippatos G., Chioncel O., Crespo Leiro M., Drozdz J., Fruhwald F., Gullestad L., Logeart D., Fabbri G., Urso R., Metra M., Parissis J., Persson H., Ponikowski P., Rauchhaus M., Voors A.A., Nielsen O.W., Zannad F., Tavazzi L. (2013). Heart Failure Association of the European Society of Cardiology (HFA), EURObservational Research Programme: regional differences and 1-year follow-up results of the Heart Failure Pilot Survey (ESC-HF Pilot). Eur. J Heart Fail..

[11] Logeart D., Isnard R., Resche-Rigon M., Seronde M.-F., de Groote P., Jondeau G., Galinier M., Mulak G., Donal E., Delahaye F., Juilliere Y., Damy T., Jourdain P., Bauer F., Eicher J.-C., Neuder Y., Trochu J.-N., on behalf of the working group on Heart Failure of the French Society of Cardiology (2013). Current aspects of the spectrum of acute heart failure syndromes in a real-life setting: the OFICA study. Eur. J. Heart Fail..

[12] von Elm E., Altman D.G., Egger M., Pocock S.J., Gøtzsche P.C., Vandenbroucke J.P. (2007). STROBE Initiative, The Strengthening the Reporting of Observational Studies in Epidemiology (STROBE) statement: guidelines for reporting observational studies. Ann. Intern. Med..

[13] White I.R., Royston P., Wood A.M. (2011). Multiple imputation using chained equations: Issues and guidance for practice. Stat. Med..

[14] Mitra R., Reiter J.P. (2016). A comparison of two methods of estimating propensity scores after multiple imputation. Stat. Methods Med. Res..

[15] Austin P.C. (2011). An introduction to propensity score methods for reducing the effects of confounding in observational studies. Multivar. Behav. Res..

[16] Kondo T., Okumura T., Matsue Y., Shiraishi A., Kagiyama N., Yamaguchi T., Kuroda S., Kida K., Mizuno A., Oishi S., Inuzuka Y., Akiyama E., Matsukawa R., Kato K., Suzuki S., Naruke T., Yoshioka K., Miyoshi T., Baba Y., Yamamoto M., Murai K., Mizutani K., Yoshida K., Kitai T., Murohara T. (2018). Specialty-related differences in the acute-phase treatment and prognosis in patients with acute heart failure – insights from REALITY-AHF -. Circ. J..

[17] Wuerz R.C., Meador S.A. (1992). Effects of prehospital medications on mortality and length of stay in congestive heart failure. Ann. Emerg. Med..

[18] Peacock W.F., Emerman C., Costanzo M.R., Diercks D.B., Lopatin M., Fonarow G.C. (2009). Early vasoactive drugs improve heart failure outcomes. Congest. Heart Fail..

[19] McCarthy M.L., Zeger S.L., Ding R., Levin S.R., Desmond J.S., Lee J., Aronsky D. (2009). Crowding delays treatment and lengthens emergency department length of stay, even among high-acuity patients. Ann. Emerg. Med..

[20] Bernstein S.L., Aronsky D., Duseja R., Epstein S., Handel D., Hwang U., McCarthy M., John McConnell K., Pines J.M., Rathlev N., Schafermeyer R., Zwemer F., Schull M., Asplin B.R. (2009). Society for academic emergency medicine, emergency department crowding task force, the effect of emergency department crowding on clinically oriented outcomes. Acad. Emerg. Med. Off. J. Soc. Acad. Emerg. Med..

[21] Ponikowski P., Voors A.A., Anker S.D., Bueno H., Cleland J.G.F., Coats A.J.S., Falk V., González-Juanatey J.R., Harjola V.-P., Jankowska E.A., Jessup M., Linde C., Nihoyannopoulos P., Parissis J.T., Pieske B., Riley J.P., Rosano G.M.C., Ruilope L.M., Ruschitzka F., Rutten F.H., van der Meer P. (2016). ESC Guidelines for the diagnosis and treatment of acute and chronic heart failure: The Task Force for the diagnosis and treatment of acute and chronic heart failure of the European Society of Cardiology (ESC)Developed with the special contribution of the Heart Failure Association (HFA) of the ESC. Eur. Heart J..

[22] M Komadja, Hospitalization for heart failure: can we prevent it? Can we predict it? 37 (2015) 119–121.

[23] Cluzol L., Cautela J., Michelet P., Roch A., Kerbaul F., Mancini J., Laine M., Peyrol M., Robin F., Paganelli F., Bonello L., Thuny F. (2017). Prehospital and in-hospital course of care for patients with acute heart failure: features and impact on prognosis in “real life”. Arch. Cardiovasc. Dis..

[24] Arrigo M., Gayat E., Parenica J., Ishihara S., Zhang J., Choi D.-J., Park J.J., Alhabib K.F., Sato N., Miro O., Maggioni A.P., Zhang Y., Spinar J., Cohen-Solal A., Iwashyna T.J., Mebazaa A. (2017). GREAT Network, precipitating factors and 90-day outcome of acute heart failure: a report from the intercontinental GREAT registry. Eur. J. Heart Fail..

[25] Zannad F., Mebazaa A., Juillière Y., Cohen-Solal A., Guize L., Alla F., Rougé P., Blin P., Barlet M.-H., Paolozzi L., Vincent C., Desnos M., Samii K. (2006). for the EFICA Investigators, clinical profile, contemporary management and one-year mortality in patients with severe acute heart failure syndromes: The EFICA study☆. Eur. J. Heart Fail..

[26] Teixeira A., Parenica J., Park J.J., Ishihara S., AlHabib K.F., Laribi S., Maggioni A., Miró Ò., Sato N., Kajimoto K., Cohen-Solal A., Fairman E., Lassus J., Mueller C., Peacock W.F., Januzzi J.L., Choi D.-J., Plaisance P., Spinar J., Mebazaa A., Gayat E., on behalf of the GREAT (Global Research on Acute Conditions Team) Network (2015). Clinical presentation and outcome by age categories in acute heart failure: results from an international observational cohort: Acute heart presentation among age categories. Eur. J. Heart Fail..

